# Ferroptosis: A potential target of macrophages in plaque vulnerability

**DOI:** 10.1515/biol-2022-0722

**Published:** 2023-10-02

**Authors:** Yu Li, Ji-Qing Ma, Chao-Chen Wang, Jian Zhou, Yu-Dong Sun, Xiao-Long Wei, Zhi-Qing Zhao

**Affiliations:** Department of Vascular Surgery, Changhai Hospital, The PLA Naval Medical University, 168 Changhai Road, Shanghai 200433, China; Department of General Surgery, Jinling Hospital, Medical School of Nanjing University, Nanjing 201411, China

**Keywords:** atherosclerosis, ferroptosis, macrophage, plaque vulnerability

## Abstract

Plaque vulnerability has been the subject of several recent studies aimed at reducing the risk of stroke and carotid artery stenosis. Atherosclerotic plaque development is a complex process involving inflammation mediated by macrophages. Plaques become more vulnerable when the equilibrium between macrophage recruitment and clearance is disturbed. Lipoperoxides, which are affected by iron levels in cells, are responsible for the cell death seen in ferroptosis. Ferroptosis results from lipoperoxide-induced mitochondrial membrane toxicity. Atherosclerosis in ApoE(−/−) mice is reduced when ferroptosis is inhibited and iron intake is limited. Single-cell sequencing revealed that a ferroptosis-related gene was substantially expressed in atherosclerosis-modeled macrophages. Since ferroptosis can be regulated, it offers hope as a non-invasive method of treating carotid plaque. In this study, we discuss the role of ferroptosis in atherosclerotic plaque vulnerability, including its mechanism, regulation, and potential future research directions.

## 1 Introduction

Ischemic cerebral strokes, a major cause of severe morbidity and mortality, are caused primarily by vulnerable atherosclerotic plaque in the carotid artery [1,2]. Ischemic stroke rates have fallen worldwide over the past decade, but they have increased dramatically in East Asia, especially China [3,4,5]. Thus, plaque vulnerability has become an increasingly central issue in the treatment and prevention of carotid artery stenosis and stroke [1,6]. Atherosclerosis complications are more strongly linked to plaque stability than to plaque size [7]. Plaque characteristics are more reliable than symptoms and stenosis for predicting strokes and choosing surgical interventions [8]. Most ischemic strokes are brought on by plaque rupture, which is characterized by a necrotic core, intraplaque hemorrhage, immunocyte infiltration, and the rupture of thin fibrous caps [9,10].

Increased macrophages relative to lipids in the plaque make the plaque more susceptible to rupture [11]. Macrophage death is linked to inflammation, oxidative stress, and lipid metabolism [12]. Macrophage death from recruitment due to pro-inflammatory factors [13], hypoxia from inadequate blood supply [14], and foam cell formation due to lipoprotein retention [15] are all hallmarks of advanced plaques. Plaque necrosis and rupture are facilitated by factors such as a necrotic core, a weakened fibrous cap, and increased inflammation. Defects in macrophage recruitment and clearance lead to these characteristics of plaque vulnerability [16,17,18].

Sullivan proposed a theory in 1981 that iron buildup was associated with the onset of cardiovascular disease [19]. However, contradictory findings have been found in clinical studies and animal experiments. Increased levels of the ubiquitous ferritin (FTN) storage protein serum FTN are not always associated with an increased risk of cardiovascular disease [20,21]. Macrophages in atherosclerotic plaques have been found to undergo ferroptosis, a form of iron-dependent cell programmed death that differs from apoptosis, cell necrosis, and autophagy, and explains the effect of iron overload on atherosclerotic vascular disease leading to cell death [22]. The systemic use of ferroptosis agents on plaques should be approached with caution, despite the fact that ferroptosis may hold promise in the treatment of cancer and other diseases[23]. Macrophage ferroptosis causes lipid peroxidation [24], inflammation [25], and phenotypic changes [26] that are all closely related to plaque, though its precise role in carotid plaque vulnerability is not well defined. Nonetheless, its role in macrophages has been shown to be crucial in determining plaque vulnerability [27]. Due to their dual role in inflammation and necrotic core, macrophage ferroptosis in plaques warrants a re-evaluation. This review provides an overview of ferroptosis and its regulatory mechanisms, discusses the link between macrophage ferroptosis and plaque vulnerability, and points researchers in a new direction in their study of carotid atherosclerotic plaques, which may ultimately lead to the discovery of novel therapeutic targets.

## 2 Macrophages in vulnerable plaques

Disparities in recruitment and clearance cause macrophages to increase plaque vulnerability [28,29], which is reflected in the aggravation of local inflammation and formation of the necrotic core [16].

### 2.1 Inflammation-associated macrophages

An initial step in the development of atherosclerosis is the release of chemokines and adhesion factors from the endothelium, which entice monocytes to bind to the endothelium [30]. Upon binding, monocytes enter the subendothelium, where they are induced by chemokines to undergo the irreversible process of macrophage differentiation and begin ingesting lipoproteins [31]. Nonetheless, macrophages are versatile and they are capable of polarization [32]. Within atherosclerotic plaques, various macrophage subtypes perform specialized phagocytic and secretory roles [33]. Macrophages migrate to the plaque because of the pro-inflammatory role played by M1 macrophages, which secrete pro-inflammatory factors to sustain the recruitment of immunocytes [34]. M1 macrophages increase the risk of plaque rupture and thrombosis by secreting pro-inflammatory factors and matrix-degrading enzymes, which in turn induce the proliferation and migration of vascular smooth muscle cell (VSMC)-derived foam cells to the intima via paracrine effects [35].

After ingesting lipoproteins, macrophages transform into foam cells [36]. Foam cells secrete extracellular matrix molecules that retain lipoproteins [37], and the resulting inflammation attracts more monocytes [38]. Meanwhile, macrophage death is the cause of necrotic core and a source of pro-inflammatory factors and damage-associated molecular patterns (DAMPs), contributing to inflammation, which in turn attracts more macrophages and leads to their aggregation and death [39]. In atherosclerosis, macrophages are responsible for secreting the DAMP HMGB1. Secretion and transcription of pro-inflammatory cytokines by NF-κB in response to HMGB1 increase plaque vulnerability. Inhibiting HMGB1 expression is one mechanism by which statins reduce plaque formation [40]. Plaque progression is hastened by inflammation that worsens over time. Consequently, stopping inflammation would have huge clinical implications.

### 2.2 Necrotic core-associated macrophages

Macrophage death has the potential to reduce inflammation, which could be helpful in the early stages of atherosclerotic plaque formation. However, necrotic core formation, thinning of the fibrous cap, and exacerbated inflammation can result from defects in the recruitment and clearance of dead macrophages, all of which are hallmarks of plaque vulnerability [16,17,18]. Thrombosis and embolization of distal arteries like the coronary, cerebrovascular, or limb arteries can occur if the intima eventually ruptures, exposing tissue factors to coagulation and increasing platelets in the blood [41]. The presence of apoptotic macrophages at the site of plaque rupture provides further evidence that macrophages play a role in the rupturing of advanced plaques. Drug-coated balloons and drug-eluting stents are two methods of preventing plaque progression and reducing the risk of plaque rupture, despite the complexity of the process.

## 3 Ferroptosis mechanisms

In 2012, Dixon et al. first identified ferroptosis, an iron-dependent form of regulated cell death mediated by elevated lipid peroxidation. Morphologically, biochemically, and genetically, this form of cell death is distinct from apoptosis, necrosis, and autophagy [42]. Mitochondria undergo morphological changes during ferroptosis, including fragmentation and cristae enlargement [42,43]. Ferroptosis, in contrast to other forms of cell death, does not share the same biochemical characteristics of energy depletion and is not regulated by the same compounds [44].

**Figure 1:**
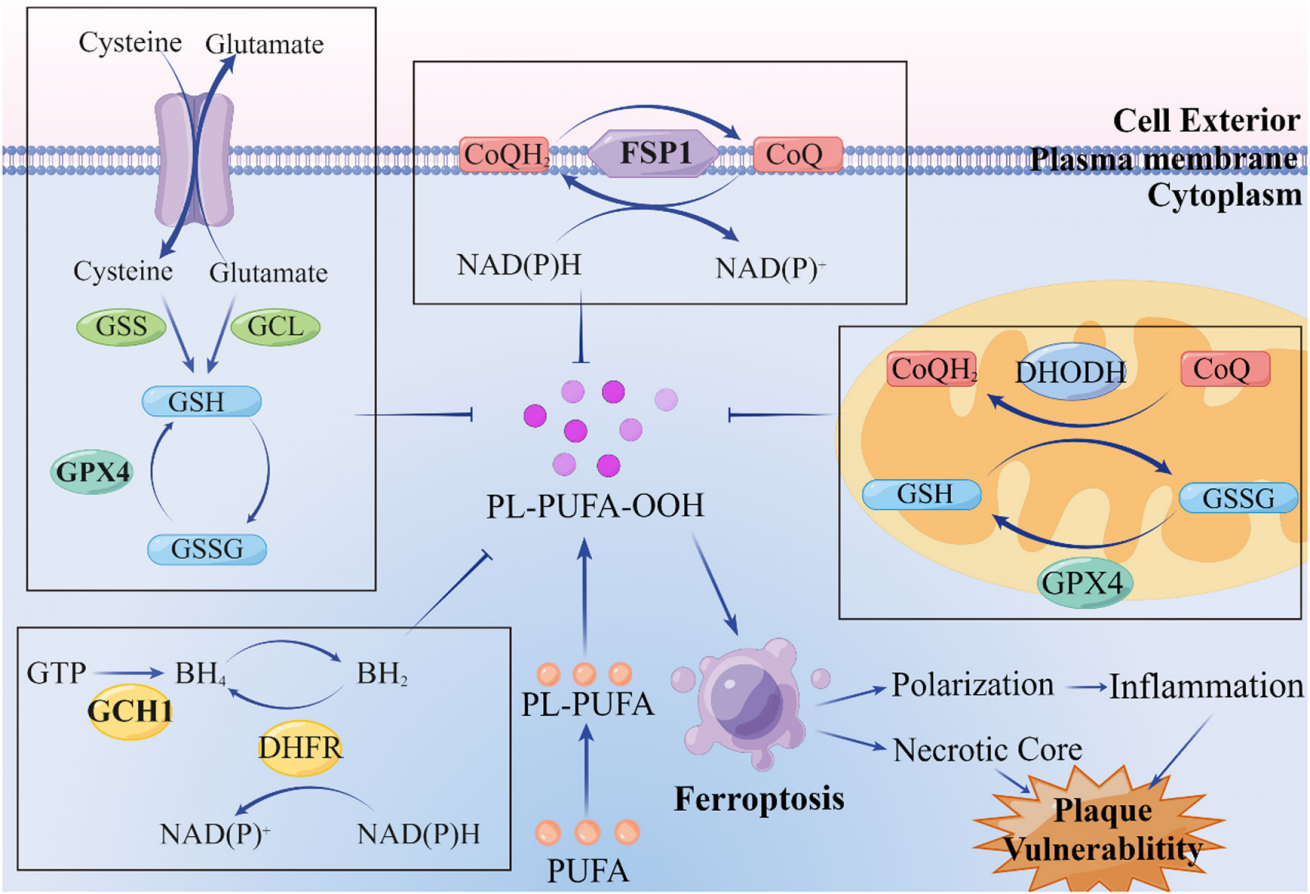
Macrophage ferroptosis regulation in atherosclerotic plaques. The SLC7A11-GSH-GPX4 and GCH1-BH4-PL cytoplasmic pathways, the FSP1-CoQ-NAD(P)H cell membrane pathway, and the DHODH-CoQ mitochondrial pathway all play roles in regulating macrophage ferroptosis.

Iron chelators were discovered to prevent cell death during a systematic study of lethal compounds, leading to the discovery of ferroptosis. Ferroptosis is strongly correlated with intracellular iron concentration [42]. Several proteins, such as transferrin receptor 1 (TFR1), divalent metal transporter 1 (DMT1), FTN, and ferroportin (FPN), regulate iron homeostasis in cells. The prevalence of ferroptosis is significantly affected by the amount of iron consumed daily. The stomach acid converts ingested Fe^3+^ to the more absorbable form Fe^2+^. Ceruloplasmin converts ferrous ion (Fe^2+^) to Fe^3+^, allowing it to bind with transferrin (TF) and be internalized via endocytosis [45]. Plasma TF is involved in the TF cycle, which transports iron from the blood, lymph, and cerebrospinal fluid to cells [46]. The enzyme ferrireductase reduces Fe^3+^ to Fe^2+^ [47]. DMT1, a transmembrane responsible for transporting various divalent metal cations into cells, then releases the unstable Fe^2+^ and transports it into the cytoplasm [48]. FTN can act as a reservoir for Fe^2+^, while FPN can secrete it [49]. FTN, a major iron storage protein, is made up of the heavy chain (FTH1) and the light chain (FTL) [50]. The iron content is regulated to maintain intracellular iron homeostasis. Iron is stored in a variety of proteins, including FTN, where it is in a form that is both non-toxic and bioavailable [51]. However, the labile iron pool (LIP) increases when the iron overload is greater than the storage capacity [52]. This causes ferroptosis by disrupting intracellular iron homeostasis, catalyzing the Fenton reaction to generate reactive oxygen species (ROS) [53], and subsequently peroxidizing polyunsaturated fatty acids [54]. Changes in mitochondrial morphology, such as mitochondrial fragmentation and cristae enlargement, are linked to the cytotoxicity of lipid peroxidation and occur during the process of ferroptosis [55] (Figure 1).

## 4 Ferroptosis regulation

### 4.1 Ferroptosis in cytoplasm

Inhibiting lipid peroxide production is central to controlling ferroptosis, which occurs because lipid peroxides are toxic to mitochondria. The production of lipid peroxides is controlled in the cytoplasm by two distinct pathways: SLC7A11-GSH-GPX4 and GCH1-BH4-PL.

Glutathione (GSH) is a tripeptide made up of glycine, cysteine, and glutamic acid that serves as the body’s primary antioxidant [56]. Loss of GSH has been linked to cell death since the 1970s [57]. GSH inhibits ROS production and keeps the cellular environment reduced [58]. SLC7A11 is a catalytic subunit of System Xc-, which regulates intracellular glutathione levels via the intercellular exchange of cysteine and glutamate [59]. Systemic Xc- is activated by the Nrf2-Keap1 pathway, and either increased expression of Nrf2 or decreased expression of Keap1 improves the resistance of ferroptosis in atherosclerosis [60]. By targeting System Xc selectively, erastin reduces GSH levels by depleting cysteine, a precursor for GSH [61]. Depletion of intracellular cysteine caused by SLC7A11 inhibition reduces GSH synthesis, which in turn reduces glutathione peroxidase 4 (GPX4) expression [62]. ATF3 inhibits SLC7A11, causing lipid peroxidation and ferroptosis by consuming GSH [63]. The GSH-dependent enzyme GPX4 regulates ferroptosis by binding to lipid hydroperoxides and reducing them to lipid alcohols in the cytosol and mitochondria (Figure 1) [64]. Overexpression of GPX4 significantly reduces lipoperoxide and atherosclerosis progression in ApoE(−/−) mice [65]. Since RSL3 inhibits GPX4 activity without causing a decrease in GSH levels, it acts through a mechanism distinct from that of erastin [66]. Notably, statins, the lipid-lowering drugs commonly used in atherosclerosis, can inhibit GPX4, a type of selenoprotein [67], leading to plaque progression [68]. By degrading hydroperoxides and lipoxygenases (LOXs), GPX4 not only acts as an intracellular antioxidant enzyme, but it also directly scavenges for phospholipid (PL) hydroperoxides on membranes [69].

The production of tetrahydrobiopterin (BH4) and dihydrobiopterin (BH2) is limited by the enzyme GTP cyclohydrolase-1 (GCH1) (Figure 1) [70]. The oxidized low-density lipoprotein (ox-LDL) present in hypercholesterolemia downregulates the mRNA expression of GCH1 and BH4, which in turn leads to NO-induced endothelial damage in atherosclerosis [71]. The involvement of GCH1 and its metabolic derivative BH4/BH2 in the regulation of ferroptosis was discovered through a genome-wide activation screen [72]. BH4 selectively inhibits PL oxidation and speeds up the production of CoQ10, both of which require dihydrofolate reductase [73].

Increased levels of BH4 caused by overexpression of GCH1 induce lipid remodeling and inhibit ferroptosis by selectively blocking phospholipid peroxidation [72]. Additionally, GCH1 protects cells from ferroptosis by regulating the oxidative balance of macrophages and BH4 [36], making it a potent antioxidant. The GCH1-BH4-PL axis regulates the endogenous generation of the antioxidant BH4 and CoQ10, as well as the peroxidation of polyunsaturated phospholipids, and is linked to ROS production as a regulator against ferroptosis [74]. In contrast to the SLC7A11-GSH-GPX4 pathway, the GCH1-BH4-PL axis is involved in ferroptosis resistance against GPX4-independent ferroptosis [72].

### 4.2 Ferroptosis in mitochondria

Mitochondrial DHODH-CoQ and SLC7A11-GSH-GPX4 have similar roles. SLC7A11-GSH-GPX4 functions similarly to its cytoplasmic counterpart.

Dihydroorotate dehydrogenase (DHODH) has been shown to inhibit ferroptosis and decrease mitochondrial lipid peroxidation [75], effects that are thought to be unrelated to its nucleotide production function [76]. DHODH catalyzes the formation of dihydroorotate and orotate (OA), and, like ferroptosis suppressor protein 1 (FSP1), prevents the production of peroxide by reducing CoQ to CoQH2 (Figure 1) [77]. DHODH substrate supplementation has been shown to have a significant effect on ferroptosis induced by GPX4 inhibition [75].

DHODH inhibition does not affect GPX4 and SLC7A11 activity or GSH content but does sensitize cells to ferroptosis inducers. Overexpression or knockout of FSP1 does not affect RSL3-induced ferroptosis in DHODH(−/−) cells. Ferroptosis is not stimulated by the inhibition of DHODH in FSP1(−/−) cells. In contrast, inhibition of DHODH makes wild-type cells more susceptible to RSL3-induced ferroptosis. Mitochondrial DHODH inhibits peroxidation similar to that of GPX4 and FSP1 [75].

### 4.3 Ferroptosis in the cell membrane

By catalyzing the reduction of ubiquinone (CoQ) to ubiquinol (CoQH2) on the cell membrane, the FSP1, also known as mitochondrial apoptosis-inducing factor 2, acts as a GSH-independent ferroptosis inhibitor (Figure 1). Inhibition of FSP1 leads to elevated lipoperoxides even in the presence of functional GPX4 [78]. An N-terminal hydrophobic sequence and an NADH oxidoreductase domain containing flavoprotein are both present in FSP1, making it an effective inhibitor of ferroptosis. To facilitate the reduction of CoQ by flavoprotein oxidoreductase, N-myristoylation of FSP1 is required in the endoplasmic reticulum, the cell membrane, and the Golgi apparatus. Inhibition of ferroptosis can be attributed to CoQH2, which acts as a free radical that recruits antioxidants on the cell membrane to prevent lipid peroxidation. Meanwhile, NAD(P)H is consumed by FSP1 to catalyze the regeneration of CoQ [79]. FSP1 knockout cells are extremely vulnerable to RSL3-induced ferroptosis, but this sensitivity can be mitigated by the presence of the potent inhibitor iFSP1. The anti-ferroptosis function of FSP1-CoQ10-NAD(P)H is independent of glutathione levels in the cell, suggesting that this antioxidant system plays a role at the membrane level [80].

## 5 Ferroptosis of macrophages in atherosclerosis

In comparison to iron accumulation in healthy arteries, iron accumulation in atherosclerotic plaques is dramatically higher [20]. There is a correlation between elevated iron levels and the development of oxidative stress, inflammation, and macrophage-derived foam cells [81]. Key features of advanced plaques include iron overload in macrophages, lipid peroxidation, intraplaque hemorrhage, and ROS accumulation [82]. Plaque formation in ApoE(−/−) mice fed a high-fat diet is significantly reduced in the presence of the ferroptosis inhibitor ferrostatin-1. By increasing the expressions of SLC7A11 and GPX4, two key regulators of iron homeostasis, ferrostatin-1 prevents iron accumulation and lipid peroxidation in mice [83]. These results provide further evidence that ferroptosis contributes to atherosclerosis. Ferroptosis may be the cause of macrophage death after recruitment, which in turn stimulates the recruitment of even more macrophages.

The increased risk of plaque rupture is a direct result of the necrotic core formation and atherosclerotic plaque progression induced by macrophage death. Macrophage ferroptosis has been linked to increased atherosclerotic plaque vulnerability. Ferroptosis is triggered by iron, and its primary mechanism is the Fenton reaction, which generates ROS and lipid peroxidation [42]. Ceruloplasmin converts Fe^2+^ into Fe^3+^ *in vivo*, and TFR1 facilitates its entry into cells [84]. Foam cells contribute to the development of human carotid plaque by having an abnormally high level of TFR1, a cell surface receptor involved in transferrin-mediated iron uptake and FTN synthesis [85]. To prevent oxidative stress from the Fe^2+^-mediated Fenton reaction and to maintain intracellular iron homeostasis and prevent ferroptosis, the major iron-storage protein with ferroxidase activity in cells, FTN, oxidizes Fe^2+^ to Fe^3+^ and stores it [86]. Decreases in FTN lead to an increase in LIP, which in turn increases ROS production and makes cells more vulnerable to ferroptosis [87]. Atherosclerotic plaques are associated with iron accumulation. Atherosclerotic plaque macrophage iron and lipid accumulation both play a role in plaque development and progression [27].

Atherosclerotic plaque vulnerability is also associated with the amount of iron present in the body. Iron chelators or dietary iron restriction slowed atherosclerosis development in ApoE(−/−) mice without influencing serum total cholesterol or triglyceride levels [20]. Through its interaction with FPN, hepcidin inhibits iron efflux in macrophages, raises intracellular iron concentration, and stimulates ferroptosis, all of which contribute to the development of inflammation and plaque [88]. Conversely, iron overload is aggravated by iron retention in macrophages via the TLR4/NF-κB pathway [89].

The mitochondrial toxicity of lipid peroxide, which results in mitochondrial changes and cell death, is the physiological basis of ferroptosis. In atherosclerosis, LOXs expressed by macrophages is a key enzyme in ROS production that promotes ferroptosis. Deposition of ox-LDL in the subendothelial space is greatly reduced by 12/15-LOX inhibition [90]. In addition to endothelial cells, foam cells, and VSMC, oxidative phospholipids (ox-PL) induced by ROS in macrophages promote plaque progression. Thus, inhibition of ox-PL in macrophages can reduce inflammation and calcification [27].

## 6 Future directions and therapeutic implications

### 6.1 Decreasing iron content

Sullivan proposed a theory in 1981 that iron content is related to cardiovascular disease [19], though he has not been able to confirm this view. However, in atherosclerotic plaques, many studies have found that iron content is higher than that of normal vascular tissue, especially in advanced plaques. Although a few studies provide direct evidence to confirm the correlation between the iron content and plaque vulnerability, this may indicate the direction of future research, that is, the detection of plaque iron content by imaging methods to determine the risk of atherosclerotic plaque complications.

After intraplaque hemorrhage, advanced atherosclerotic plaques have a higher iron content because of the necrotic core and macrophage infiltration [91]. Iron not only accumulates in local plaques but also affects plaque progression via serum iron content [20]. Overloading FTN causes iron homeostasis to be disrupted, leading to an increase in LIP, which is the first step in ferroptosis [92]. Thus, effectively regulating iron content in cells can affect plaques through ferroptosis [93]. Elevated serum FTN concentration, along with high levels of free iron and transferrin saturation, has been linked to an increased risk of cardiovascular disease in humans [94]. Elevated intracellular iron content due to hepcidin enhances inflammation and progression of atherosclerotic plaques [88]. Iron chelators and dietary restrictions slowed plaque progression and decreased inflammation and vascular injury in ApoE(−/−) mice [20]. Heat shock proteins (HSPs) have been found to downregulate TFR1-mediated iron uptake and thereby reduce intracellular iron concentration [95]. HSPs have been reported to inhibit inflammation and the progression of plaques [96], and their role in macrophages provides a direction for future research on plaque stability.

Iron chelators are the most common pharmaceutical agents for reducing iron accumulation [97]. They can penetrate cell membranes and bind to free ferrous iron, thereby preventing the Fenton reaction [98]. Iron chelators are also effective in decreasing LIP and preventing ferroptosis in cancer and neurodegenerative diseases [99,100]. Desferrioxamine, deferiprone, and deferasirox are types of iron chelators that have been found to reduce iron concentrations [101]. An increase in LIP can lead to the progression of atherosclerosis and the accumulation of immune cells [102]. When used together, iron chelators, iron-restricted diets, and anti-inflammatory therapies can inhibit iron-overload-related atherosclerosis [20].

Atherosclerosis risk factors include increased intracellular iron content, which promotes inflammation, lipid oxidation, and necrotic core. By reducing the lipid content, Statins have been shown to be effective in delaying plaque progression but not pathology progression. It is proved that iron content can affect plaque vulnerability by causing ferroptosis in plaque, so reducing blood iron content to improve plaque stability has a good development prospect. Inhibiting ferroptosis, atherosclerosis, and plaque vulnerability can be achieved by reducing intracellular iron content. Although there is currently no concrete evidence for this, it has the potential to be a useful treatment in the near future.

### 6.2 Inflammation and polarization

Inflammation in the plaque is the main cause of rapid plaque progression, necrosis, and rupture of advanced plaque. At present, researchers need an exact and comprehensive understanding of the causes of inflammation in plaques. Although it has been recognized that macrophage death plays a key role, there are many causes of macrophage death including apoptosis, necrosis, pyroptosis, ferroptosis, and other death modes. Delaying plaque progression and decreasing plaque vulnerability through ferroptosis intervention will be a new direction for the stability of atherosclerotic plaques in the future.

The polarization of macrophages is a crucial factor of plaque formation and a potential target for atherosclerotic plaque vulnerability [34]. M2 macrophages have strong phagocytosis and anti-inflammation effects and can transform M1 macrophages into M2 macrophages by initiating anti-inflammatory signal transduction [103]. M2 macrophages play a role in tissue repair by activating extracellular matrix synthesis and VSMC proliferation, thereby reducing vulnerability [104]. The role of NO synthase, which inhibits lipid peroxidation in place of GPX4, is associated with the sensitivity of RSL3-induced macrophage ferroptosis in various subtypes [105]. More research is needed on the function of M2 macrophages in preventing ferroptosis, reducing inflammation, preventing necrotic core formation, and enhancing plaque stability. Further, in advanced atherosclerosis, ferroptosis promotes plaque instability, so a better understanding of the molecular and cellular mechanisms involved is necessary.

Although this review focuses on inflammation, oxidative stress, and polarization, the interplay between these molecular effectors remains unclear. Clarifying the mechanism by which defects in macrophages cause ferroptosis and identifying the cellular effectors that are defective are fundamental. Several lines of evidence point to macrophage ferroptosis as a major contributor to atherosclerotic plaque vulnerability. Although the role of ferroptosis in atherosclerosis has been investigated previously, researchers are now focusing on plaque vulnerability instead. Further studies on the correlation between macrophage ferroptosis and plaque vulnerability can provide new insights and therapeutic strategies for stroke prevention and atherosclerotic plaques.

## 7 Summary and conclusion

Carotid plaque vulnerability is a major factor contributing to cerebral stroke, a leading cause of severe morbidity and mortality. Thus, investigating the molecular mechanisms underlying plaque vulnerability is essential. In this review, we discussed the most up-to-date findings on ferroptosis and provided evidence that iron overload-induced ferroptosis of macrophages occurs in advanced atherosclerotic plaques, resulting in alterations in inflammation and oxidative stress that impact plaque stability. In the future, research on iron content, inflammation, and macrophage polarization of ferroptosis may provide new insights into plaque vulnerability. While ferroptosis may play a role in at least one of the many mechanisms that lead to the destabilization of atherosclerotic plaques, it is important to remember that this is itself a complex process involving the accumulation and defective resolution of inflammation. However, inhibiting ferroptosis may be an effective new strategy for treating carotid plaque. Given the high prevalence of stroke and its associated morbidity and mortality, even incremental improvements in this field could have far-reaching consequences.

## References

[1] Saba L, Saam T, Jäger HR, Yuan C, Hatsukami TS, Saloner D, et al. Imaging biomarkers of vulnerable carotid plaques for stroke risk prediction and their potential clinical implications. Lancet Neurol. 2019 Jun;18(6):559–72. 10.1016/S1474-4422(19)30035-3. 30954372

[2] van Dam-Nolen DHK, Truijman MTB, van der Kolk AG, Liem MI, Schreuder FHBM, Boersma E, et al. Carotid plaque characteristics predict recurrent ischemic stroke and TIA: The PARISK (Plaque At RISK) Study. JACC Cardiovasc Imaging. 2022 Oct;15(10):1715–26. 10.1016/j.jcmg.2022.04.003. 36202450

[3] GBD 2019 Stroke Collaborators. Global, regional, and national burden of stroke and its risk factors, 1990-2019: a systematic analysis for the Global Burden of Disease Study 2019. Lancet Neurol. 2021 Oct;20(10):795–820. 10.1016/S1474-4422(21)00252-0. PMC8443449 34487721

[4] Huang K, Liang F, Yang X, Liu F, Li J, Xiao Q, et al. Long term exposure to ambient fine particulate matter and incidence of stroke: prospective cohort study from the China-PAR project. BMJ. 2019 Dec 30;367:l6720. 10.1136/bmj.l6720. PMC7190010 31888885

[5] Wang W, Jiang B, Sun H, Ru X, Sun D, Wang L, et al. Prevalence, incidence, and mortality of stroke in China: Results from a nationwide population-based survey of 480 687 Adults. Circulation. 2017 Feb 21;135(8):759–71. 10.1161/CIRCULATIONAHA.116.025250. 28052979

[6] Saba L, Moody AR, Saam T, Kooi ME, Wasserman BA, Staub D, et al. Vessel wall-imaging biomarkers of carotid plaque vulnerability in stroke prevention trials: a viewpoint from the carotid imaging consensus group. JACC Cardiovasc Imaging. 2020 Nov;13(11):2445–56. 10.1016/j.jcmg.2020.07.046. 33153534

[7] Kopczak A, Schindler A, Sepp D, Bayer-Karpinska A, Malik R, Koch ML, et al. Complicated carotid artery plaques and risk of recurrent ischemic stroke or TIA. J Am Coll Cardiol. 2022 Jun 7;79(22):2189–99. 10.1016/j.jacc.2022.03.376. 35523659

[8] Shishikura D, Kataoka Y, Di Giovanni G, Takata K, Scherer DJ, Andrews J, et al. Progression of ultrasound plaque attenuation and low echogenicity associates with major adverse cardiovascular events. Eur Heart J. 2020 Aug 14;41(31):2965–73. 10.1093/eurheartj/ehaa173. 32243512

[9] Flaherty ML, Kissela B, Khoury JC, Alwell K, Moomaw CJ, Woo D, et al. Carotid artery stenosis as a cause of stroke. Neuroepidemiology. 2013;40(1):36–41. 10.1159/000341410. PMC3626492 23075828

[10] Hong MK, Mintz GS, Lee CW, Kim YH, Lee SW, Song JM, et al. Comparison of coronary plaque rupture between stable angina and acute myocardial infarction: a three-vessel intravascular ultrasound study in 235 patients. Circulation. 2004 Aug 24;110(8):928–33. 10.1161/01.CIR.0000139858.69915.2E. 15313951

[11] Shami A, Edsfeldt A, Bengtsson E, Nilsson J, Shore AC, Natali A, et al. Soluble CD40 Levels in plasma are associated with cardiovascular disease and in carotid plaques with a vulnerable phenotype. J Stroke. 2021 Sep;23(3):367–76. 10.5853/jos.2021.00178. PMC8521258 34649381

[12] Robinson N, Ganesan R, Hegedűs C, Kovács K, Kufer TA, Virág L. Programmed necrotic cell death of macrophages: Focus on pyroptosis, necroptosis, and parthanatos. Redox Biol. 2019 Sep;26:101239. 10.1016/j.redox.2019.101239. PMC6582207 31212216

[13] Fadini GP, Simoni F, Cappellari R, Vitturi N, Galasso S, Vigili de Kreutzenberg S, et al. Pro-inflammatory monocyte-macrophage polarization imbalance in human hypercholesterolemia and atherosclerosis. Atherosclerosis. 2014 Dec;237(2):805–8. 10.1016/j.atherosclerosis.2014.10.106. Epub 2014 Nov 4 PMID: 25463124. 25463124

[14] Hultén LM, Levin M. The role of hypoxia in atherosclerosis. Curr Opin Lipidol. 2009 Oct;20(5):409–14. 10.1097/MOL.0b013e3283307be8. 19644366

[15] Skålén K, Gustafsson M, Rydberg EK, Hultén LM, Wiklund O, Innerarity TL. Subendothelial retention of atherogenic lipoproteins in early atherosclerosis. Nature. 2002 Jun 13;417(6890):750–4. 10.1038/nature00804. 12066187

[16] Karunakaran D, Geoffrion M, Wei L, Gan W, Richards L, Shangari P, et al. Targeting macrophage necroptosis for therapeutic and diagnostic interventions in atherosclerosis. Sci Adv. 2016 Jul 22;2(7):e1600224. 10.1126/sciadv.1600224. PMC4985228 27532042

[17] Tomaniak M, Katagiri Y, Modolo R, de Silva R, Khamis RY, Bourantas CV, et al. Vulnerable plaques and patients: state-of-the-art. Eur Heart J. 2020 Aug 14;41(31):2997–3004. 10.1093/eurheartj/ehaa227. PMC8453282 32402086

[18] Lüscher TF. Inflammation and features of the vulnerable plaque: from mechanisms and imaging to outcomes. Eur Heart J. 2020 Aug 14;41(31):2923–7. 10.1093/eurheartj/ehaa686. 33216918

[19] Sullivan JL. Iron and the sex difference in heart disease risk. Lancet. 1981 Jun 13;1(8233):1293–4. 10.1016/s0140-6736(81)92463-6. 6112609

[20] Vinchi F, Porto G, Simmelbauer A, Altamura S, Passos ST, Garbowski M, et al. Atherosclerosis is aggravated by iron overload and ameliorated by dietary and pharmacological iron restriction. Eur Heart J. 2020 Jul 21;41(28):2681–95. 10.1093/eurheartj/ehz112. 30903157

[21] Khan SU, Khan MU, Riaz H, Valavoor S, Zhao D, Vaughan L, et al. Effects of nutritional supplements and dietary interventions on cardiovascular outcomes: An umbrella review and evidence map. Ann Intern Med. 2019 Aug 6;171(3):190–8. 10.7326/M19-0341. Epub 2019 Jul 9. Erratum in: Ann Intern Med. 2020 Jan 7;172(1):75–76. PMC7261374 31284304

[22] Li M, Xin S, Gu R, Zheng L, Hu J, Zhang R, et al. Novel diagnostic biomarkers related to oxidative stress and macrophage ferroptosis in atherosclerosis. Oxid Med Cell Longev. 2022 Aug 5;2022:8917947. 10.1155/2022/8917947. PMC9410850 36035208

[23] Stockwell BR, Jiang X, Gu W. Emerging Mechanisms and Disease Relevance of Ferroptosis. Trends Cell Biol. 2020 Jun;30(6):478–90. 10.1016/j.tcb.2020.02.009. PMC7230071 32413317

[24] Yang WS, Stockwell BR. Ferroptosis: Death by lipid peroxidation. Trends Cell Biol. 2016 Mar;26(3):165–76. 10.1016/j.tcb.2015.10.014. PMC4764384 26653790

[25] Chen X, Kang R, Kroemer G, Tang D. Ferroptosis in infection, inflammation, and immunity. J Exp Med. 2021 Jun 7;218(6):e20210518. 10.1084/jem.20210518. PMC8126980 33978684

[26] Dai E, Han L, Liu J, Xie Y, Kroemer G, Klionsky DJ, et al. Autophagy-dependent ferroptosis drives tumor-associated macrophage polarization via release and uptake of oncogenic KRAS protein. Autophagy. 2020 Nov;16(11):2069–83. 10.1080/15548627.2020.1714209. PMC7595620 31920150

[27] Ouyang S, You J, Zhi C, Li P, Lin X, Tan X, et al. Ferroptosis: the potential value target in atherosclerosis. Cell Death Dis. 2021 Aug 10;12(8):782. 10.1038/s41419-021-04054-3. PMC8355346 34376636

[28] Moore KJ, Koplev S, Fisher EA, Tabas I, Björkegren JLM, Doran AC, et al. Macrophage trafficking, inflammatory resolution, and genomics in atherosclerosis: JACC macrophage in CVD series (Part 2). J Am Coll Cardiol. 2018 Oct 30;72(18):2181–97. 10.1016/j.jacc.2018.08.2147. PMC6522246 30360827

[29] Tabas I. Macrophage death and defective inflammation resolution in atherosclerosis. Nat Rev Immunol. 2010 Jan;10(1):36–46. 10.1038/nri2675. PMC2854623 19960040

[30] Tedgui A, Mallat Z. Cytokines in atherosclerosis: pathogenic and regulatory pathways. Physiol Rev. 2006 Apr;86(2):515–81. 10.1152/physrev.00024.2005. 16601268

[31] Groh L, Keating ST, Joosten LAB, Netea MG, Riksen NP. Monocyte and macrophage immunometabolism in atherosclerosis. Semin Immunopathol. 2018 Feb;40(2):203–14. 10.1007/s00281-017-0656-7. PMC5809534 28971272

[32] Colin S, Chinetti-Gbaguidi G, Staels B. Macrophage phenotypes in atherosclerosis. Immunol Rev. 2014 Nov;262(1):153–66. 10.1111/imr.12218. 25319333

[33] Jinnouchi H, Guo L, Sakamoto A, Torii S, Sato Y, Cornelissen A, et al. Diversity of macrophage phenotypes and responses in atherosclerosis. Cell Mol Life Sci. 2020 May;77(10):1919–32. 10.1007/s00018-019-03371-3. PMC11104939 31720740

[34] Shapouri-Moghaddam A, Mohammadian S, Vazini H, Taghadosi M, Esmaeili SA, Mardani F, et al. Macrophage plasticity, polarization, and function in health and disease. J Cell Physiol. 2018 Sep;233(9):6425–40. 10.1002/jcp.26429. PMC13482560 29319160

[35] Grootaert MOJ, Bennett MR. Vascular smooth muscle cells in atherosclerosis: time for a re-assessment. Cardiovasc Res. 2021 Sep 28;117(11):2326–39. 10.1093/cvr/cvab046. PMC8479803 33576407

[36] McNeill E, Crabtree MJ, Sahgal N, Patel J, Chuaiphichai S, Iqbal AJ, et al. Regulation of iNOS function and cellular redox state by macrophage Gch1 reveals specific requirements for tetrahydrobiopterin in NRF2 activation. Free Radic Biol Med. 2015 Feb;79:206–16. 10.1016/j.freeradbiomed.2014.10.575. PMC4344222 25451639

[37] Bekkering S, Quintin J, Joosten LA, van der Meer JW, Netea MG, Riksen NP. Oxidized low-density lipoprotein induces long-term proinflammatory cytokine production and foam cell formation via epigenetic reprogramming of monocytes. Arterioscler Thromb Vasc Biol. 2014 Aug;34(8):1731–8. 10.1161/ATVBAHA.114.303887. 24903093

[38] Hansson GK, Robertson AK, Söderberg-Nauclér C. Inflammation and atherosclerosis. Annu Rev Pathol. 2006;1:297–329. 10.1146/annurev.pathol.1.110304.100100. 18039117

[39] Roh JS, Sohn DH. Damage-associated molecular patterns in inflammatory diseases. Immune Netw. 2018 Aug 13;18(4):e27. 10.4110/in.2018.18.e27. PMC6117512 30181915

[40] Liu M, Yu Y, Jiang H, Zhang L, Zhang PP, Yu P, et al. Simvastatin suppresses vascular inflammation and atherosclerosis in ApoE(-/-) mice by downregulating the HMGB1-RAGE axis. Acta Pharmacol Sin. 2013 Jun;34(6):830–6. 10.1038/aps.2013.8. PMC4002903 23564080

[41] Babaniamansour P, Mohammadi M, Babaniamansour S, Aliniagerdroudbari E. The relation between atherosclerosis plaque composition and plaque rupture. J Med Signals Sens. 2020 Nov 11;10(4):267–73. 10.4103/jmss.JMSS_48_19. PMC7866947 33575199

[42] Dixon SJ, Lemberg KM, Lamprecht MR, Skouta R, Zaitsev EM, Gleason CE, et al. Ferroptosis: an iron-dependent form of nonapoptotic cell death. Cell. 2012 May 25;149(5):1060–72. 10.1016/j.cell.2012.03.042. PMC3367386 22632970

[43] Doll S, Proneth B, Tyurina YY, Panzilius E, Kobayashi S, Ingold I, et al. ACSL4 dictates ferroptosis sensitivity by shaping cellular lipid composition. Nat Chem Biol. 2017 Jan;13(1):91–8. 10.1038/nchembio.2239. PMC5610546 27842070

[44] Zheng J, Conrad M. The metabolic underpinnings of ferroptosis. Cell Metab. 2020 Dec 1;32(6):920–37. 10.1016/j.cmet.2020.10.011. 33217331

[45] El Hage Chahine JM, Hémadi M, Ha-Duong NT. Uptake and release of metal ions by transferrin and interaction with receptor 1. Biochim Biophys Acta. 2012 Mar;1820(3):334–47. 10.1016/j.bbagen.2011.07.008. 21872645

[46] Yu Z, Zheng X, Wang C, Chen C, Ning N, Peng D, et al. The traditional Chinese medicine Hua tuo Zai Zao wan alleviates atherosclerosis by deactivation of inflammatory macrophages. Evid Based Complement Alternat Med. 2022 Mar 28;2022:2200662. 10.1155/2022/2200662. PMC8979684 35388302

[47] McKie AT, Latunde-Dada GO, Miret S, McGregor JA, Anderson GJ, Vulpe CD, et al. Molecular evidence for the role of a ferric reductase in iron transport. Biochem Soc Trans. 2002 Aug;30(4):722–4. 10.1042/bst0300722. 12196176

[48] Garrick MD, Dolan KG, Horbinski C, Ghio AJ, Higgins D, Porubcin M, et al. DMT1: a mammalian transporter for multiple metals. Biometals. 2003 Mar;16(1):41–54. 10.1023/a:1020702213099. 12572663

[49] Zhang M, Nakamura K, Kageyama S, Lawal AO, Gong KW, Bhetraratana M, et al. Myeloid HO-1 modulates macrophage polarization and protects against ischemia-reperfusion injury. JCI Insight. 2018 Oct 4;3(19):e120596. 10.1172/jci.insight.120596. PMC6237471 30282830

[50] Muhoberac BB, Vidal R. Iron, ferritin, hereditary ferritinopathy, and neurodegeneration. Front Neurosci. 2019 Dec 11;13:1195. 10.3389/fnins.2019.01195. PMC6917665 31920471

[51] Theil EC. Iron homeostasis and nutritional iron deficiency. J Nutr. 2011 Apr 1;141(4):724S–8S. 10.3945/jn.110.127639. PMC3056584 21346101

[52] Imoto S, Sawamura T, Shibuya Y, Kono M, Ohbuchi A, Suzuki T, et al. Labile iron, ROS, and cell death are prominently induced by haemin, but not by non-transferrin-bound iron. Transfus Apher Sci. 2022 Apr;61(2):103319. 10.1016/j.transci.2021.103319. 34801431

[53] Bystrom LM, Guzman ML, Rivella S. Iron and reactive oxygen species: friends or foes of cancer cells? Antioxid Redox Signal. 2014 Apr 20;20(12):1917–24. 10.1089/ars.2012.5014. PMC3967355 23198911

[54] Stockwell BR, Friedmann Angeli JP, Bayir H, Bush AI, Conrad M, Dixon SJ, et al. Ferroptosis: A regulated cell death nexus linking metabolism, redox biology, and disease. Cell. 2017 Oct 5;171(2):273–85. 10.1016/j.cell.2017.09.021. PMC5685180 28985560

[55] Bock FJ, Tait SWG. Mitochondria as multifaceted regulators of cell death. Nat Rev Mol Cell Biol. 2020 Feb;21(2):85–100. 10.1038/s41580-019-0173-8. 31636403

[56] Forman HJ, Zhang H, Rinna A. Glutathione: overview of its protective roles, measurement, and biosynthesis. Mol Aspects Med. 2009 Feb-Apr;30(1–2):1–12. 10.1016/j.mam.2008.08.006. PMC2696075 18796312

[57] Spielberg SP, Gordon GB. Nitrofurantoin cytotoxicity. In vitro assessment of risk based on glutathione metabolism. J Clin Invest. 1981 Jan;67(1):37–41. 10.1172/JCI110030. PMC371569 7451657

[58] Zhou J, Li XY, Liu YJ, Feng J, Wu Y, Shen HM, et al. Full-coverage regulations of autophagy by ROS: from induction to maturation. Autophagy. 2022 Jun;18(6):1240–55. 10.1080/15548627.2021.1984656. PMC9225210 34662529

[59] Conrad M, Sato H. The oxidative stress-inducible cystine/glutamate antiporter, system x (c) (-): cystine supplier and beyond. Amino Acids. 2012 Jan;42(1):231–46. 10.1007/s00726-011-0867-5. 21409388

[60] Fan Z, Wirth AK, Chen D, Wruck CJ, Rauh M, Buchfelder M, et al. Nrf2-Keap1 pathway promotes cell proliferation and diminishes ferroptosis. Oncogenesis. 2017 Aug 14;6(8):e371. 10.1038/oncsis.2017.65. PMC5608917 28805788

[61] Zhang Y, Tan H, Daniels JD, Zandkarimi F, Liu H, Brown LM, et al. Imidazole ketone erastin induces ferroptosis and slows tumor growth in a mouse Lymphoma model. Cell Chem Biol. 2019 May 16;26(5):623–33.e9. 10.1016/j.chembiol.2019.01.008. PMC6525071 30799221

[62] Yang WS, SriRamaratnam R, Welsch ME, Shimada K, Skouta R, Viswanathan VS, et al. Regulation of ferroptotic cancer cell death by GPX4. Cell. 2014 Jan 16;156(1–2):317–31. 10.1016/j.cell.2013.12.010. PMC4076414 24439385

[63] Wang L, Liu Y, Du T, Yang H, Lei L, Guo M, et al. ATF3 promotes erastin-induced ferroptosis by suppressing system Xc. Cell Death Differ. 2020 Feb;27(2):662–75. 10.1038/s41418-019-0380-z. PMC7206049 31273299

[64] Carlson BA, Tobe R, Yefremova E, Tsuji PA, Hoffmann VJ, Schweizer U, et al. Glutathione peroxidase 4 and vitamin E cooperatively prevent hepatocellular degeneration. Redox Biol. 2016 Oct;9:22–31. 10.1016/j.redox.2016.05.003. PMC4900515 27262435

[65] Guo Z, Ran Q, Roberts 2nd LJ, Zhou L, Richardson A, Sharan C, et al. Suppression of atherogenesis by overexpression of glutathione peroxidase-4 in apolipoprotein E-deficient mice. Free Radic Biol Med. 2008 Feb 1;44(3):343–52. 10.1016/j.freeradbiomed.2007.09.009. PMC2245803 18215741

[66] Imai H, Matsuoka M, Kumagai T, Sakamoto T, Koumura T. Lipid peroxidation-dependent cell death regulated by GPx4 and ferroptosis. Curr Top Microbiol Immunol. 2017;403:143–70. 10.1007/82_2016_508. 28204974

[67] Strauss E, Tomczak J, Staniszewski R, Oszkinis G. Associations and interactions between variants in selenoprotein genes, selenoprotein levels and the development of abdominal aortic aneurysm, peripheral arterial disease, and heart failure. PLoS One. 2018 Sep 6;13(9):e0203350. 10.1371/journal.pone.0203350. PMC6126836 30188935

[68] Kromer A, Moosmann B. Statin-induced liver injury involves cross-talk between cholesterol and selenoprotein biosynthetic pathways. Mol Pharmacol. 2009 Jun;75(6):1421–9. 10.1124/mol.108.053678. 19332511

[69] Imai H, Nakagawa Y. Biological significance of phospholipid hydroperoxide glutathione peroxidase (PHGPx, GPx4) in mammalian cells. Free Radic Biol Med. 2003 Jan 15;34(2):145–69. 10.1016/s0891-5849(02)01197-8. 12521597

[70] Yoneyama T, Hatakeyama K. Ligand binding to the inhibitory and stimulatory GTP cyclohydrolase I/GTP cyclohydrolase I feedback regulatory protein complexes. Protein Sci. 2001 Apr;10(4):871–8. 10.1110/ps.38501. PMC2373977 11274478

[71] Ning DS, Ma J, Peng YM, Li Y, Chen YT, Li SX, et al. Apolipoprotein A-I mimetic peptide inhibits atherosclerosis by increasing tetrahydrobiopterin via regulation of GTP-cyclohydrolase 1 and reducing uncoupled endothelial nitric oxide synthase activity. Atherosclerosis. 2021 Jul;328:83–91. 10.1016/j.atherosclerosis.2021.05.019. 34118596

[72] Kraft VAN, Bezjian CT, Pfeiffer S, Ringelstetter L, Müller C, Zandkarimi F, et al. GTP Cyclohydrolase 1/Tetrahydrobiopterin Counteract Ferroptosis through Lipid Remodeling. ACS Cent Sci. 2020 Jan 22;6(1):41–53. 10.1021/acscentsci.9b01063. PMC6978838 31989025

[73] Soula M, Weber RA, Zilka O, Alwaseem H, La K, Yen F, et al. Metabolic determinants of cancer cell sensitivity to canonical ferroptosis inducers. Nat Chem Biol. 2020 Dec;16(12):1351–60. 10.1038/s41589-020-0613-y. PMC8299533 32778843

[74] Xu L, Liu Y, Chen X, Zhong H, Wang Y. Ferroptosis in life: To be or not to be. Biomed Pharmacother. 2023 Mar;159:114241. 10.1016/j.biopha.2023.114241. 36634587

[75] Mao C, Liu X, Zhang Y, Lei G, Yan Y, Lee H, et al. DHODH-mediated ferroptosis defence is a targetable vulnerability in cancer. Nature. 2021 May;593(7860):586–90. 10.1038/s41586-021-03539-7. Epub 2021 May 12. Erratum in: Nature. 2021 Aug;596(7873):E13. PMC8895686 33981038

[76] Martínez-Reyes I, Cardona LR, Kong H, Vasan K, McElroy GS, Werner M, et al. Mitochondrial ubiquinol oxidation is necessary for tumour growth. Nature. 2020 Sep;585(7824):288–92. 10.1038/s41586-020-2475-6. PMC7486261 32641834

[77] Baldwin J, Farajallah AM, Malmquist NA, Rathod PK, Phillips MA. Malarial dihydroorotate dehydrogenase. Substrate and inhibitor specificity. J Biol Chem. 2002 Nov 1;277(44):41827–34. 10.1074/jbc.M206854200. 12189151

[78] Bersuker K, Hendricks JM, Li Z, Magtanong L, Ford B, Tang PH, et al. The CoQ oxidoreductase FSP1 acts parallel to GPX4 to inhibit ferroptosis. Nature. 2019 Nov;575(7784):688–92. 10.1038/s41586-019-1705-2. PMC6883167 31634900

[79] Nguyen HP, Yi D, Lin F, Viscarra JA, Tabuchi C, Ngo K, et al. Aifm2, a NADH Oxidase, Supports Robust Glycolysis and Is Required for Cold- and Diet-Induced Thermogenesis. Mol Cell. 2020 Feb 6;77(3):600–17.e4. 10.1016/j.molcel.2019.12.002. PMC7031813 31952989

[80] Doll S, Freitas FP, Shah R, Aldrovandi M, da Silva MC, Ingold I, et al. FSP1 is a glutathione-independent ferroptosis suppressor. Nature. 2019 Nov;575(7784):693–8. 10.1038/s41586-019-1707-0. 31634899

[81] Lapenna D, Pierdomenico SD, Ciofani G, Ucchino S, Neri M, Giamberardino MA, et al. Association of body iron stores with low molecular weight iron and oxidant damage of human atherosclerotic plaques. Free Radic Biol Med. 2007 Feb 15;42(4):492–8. 10.1016/j.freeradbiomed.2006.11.014. 17275681

[82] Vinchi F. Non-Transferrin-Bound Iron in the Spotlight: Novel Mechanistic Insights into the Vasculotoxic and Atherosclerotic Effect of Iron. Antioxid Redox Signal. 2021 Aug 20;35(6):387–414. 10.1089/ars.2020.8167. PMC8328045 33554718

[83] Bai T, Li M, Liu Y, Qiao Z, Wang Z. Inhibition of ferroptosis alleviates atherosclerosis through attenuating lipid peroxidation and endothelial dysfunction in mouse aortic endothelial cell. Free Radic Biol Med. 2020 Nov 20;160:92–102. 10.1016/j.freeradbiomed.2020.07.026. 32768568

[84] Fisher AL, Wang CY, Xu Y, Joachim K, Xiao X, Phillips S, et al. Functional role of endothelial transferrin receptor 1 in iron sensing and homeostasis. Am J Hematol. 2022 Dec;97(12):1548–59. 10.1002/ajh.26716. PMC9662186 36069607

[85] Li W, Xu LH, Forssell C, Sullivan JL, Yuan XM. Overexpression of transferrin receptor and ferritin related to clinical symptoms and destabilization of human carotid plaques. Exp Biol Med (Maywood). 2008 Jul;233(7):818–26. 10.3181/0711-RM-320. 18445768

[86] Gao F, Chen X, Xu B, Luo Z, Liang Y, Fang S, et al. Inhibition of MicroRNA-92 alleviates atherogenesis by regulation of macrophage polarization through targeting KLF4. J Cardiol. 2022 Mar;79(3):432–8. 10.1016/j.jjcc.2021.10.015. 34750028

[87] Du J, Wang T, Li Y, Zhou Y, Wang X, Yu X, et al. DHA inhibits proliferation and induces ferroptosis of leukemia cells through autophagy dependent degradation of ferritin. Free Radic Biol Med. 2019 Feb 1;131:356–69. 10.1016/j.freeradbiomed.2018.12.011. 30557609

[88] Wunderer F, Traeger L, Sigurslid HH, Meybohm P, Bloch DB, Malhotra R. The role of hepcidin and iron homeostasis in atherosclerosis. Pharmacol Res. 2020 Mar;153:104664. 10.1016/j.phrs.2020.104664. PMC7066581 31991168

[89] Kang DY, Sp N, Jo ES, Lee JM, Jang KJ. New insights into the pivotal role of iron/heme metabolism in TLR4/NF-κB signaling-mediated inflammatory responses in human monocytes. Cells. 2021 Sep 27;10(10):2549. 10.3390/cells10102549. PMC8534183 34685529

[90] Cyrus T, Witztum JL, Rader DJ, Tangirala R, Fazio S, Linton MF, et al. Disruption of the 12/15-lipoxygenase gene diminishes atherosclerosis in apo E-deficient mice. J Clin Invest. 1999 Jun;103(11):1597–604. 10.1172/JCI5897. PMC408369 10359569

[91] Kolodgie FD, Gold HK, Burke AP, Fowler DR, Kruth HS, Weber DK, et al. Intraplaque hemorrhage and progression of coronary atheroma. N Engl J Med. 2003 Dec 11;349(24):2316–25. 10.1056/NEJMoa035655. 14668457

[92] Zhang N, Yu X, Song L, Xiao Z, Xie J, Xu H. Ferritin confers protection against iron-mediated neurotoxicity and ferroptosis through iron chelating mechanisms in MPP + -induced MES23.5 dopaminergic cells. Free Radic Biol Med. 2022 Nov 20;193(Pt 2):751–63. 10.1016/j.freeradbiomed.2022.11.018. 36395957

[93] Fernández-García V, González-Ramos S, Avendaño-Ortiz J, Martín-Sanz P, Delgado C, Castrillo A, et al. NOD1 splenic activation confers ferroptosis protection and reduces macrophage recruitment under pro-atherogenic conditions. Biomed Pharmacother. 2022 Apr;148:112769. 10.1016/j.biopha.2022.112769. 35247718

[94] Fang X, Ardehali H, Min J, Wang F. The molecular and metabolic landscape of iron and ferroptosis in cardiovascular disease. Nat Rev Cardiol. 2023 Jan;20(1):7–23. 10.1038/s41569-022-00735-4. PMC9252571 35788564

[95] Chen H, Zheng C, Zhang Y, Chang YZ, Qian ZM, Shen X. Heat shock protein 27 downregulates the transferrin receptor 1-mediated iron uptake. Int J Biochem Cell Biol. 2006;38(8):1402–16. 10.1016/j.biocel.2006.02.006. 16546437

[96] Frostegård J. Immunity, atherosclerosis and cardiovascular disease. BMC Med. 2013 May 1;11:117. 10.1186/1741-7015-11-117. PMC3658954 23635324

[97] Hatcher HC, Singh RN, Torti FM, Torti SV. Synthetic and natural iron chelators: therapeutic potential and clinical use. Future Med Chem. 2009 Dec;1(9):1643–70. 10.4155/fmc.09.121. PMC3821171 21425984

[98] Terman A, Kurz T. Lysosomal iron, iron chelation, and cell death. Antioxid Redox Signal. 2013 Mar 10;18(8):888–98. 10.1089/ars.2012.4885. 22909065

[99] Lokesh KN, Raichur AM. Bioactive nutraceutical ligands and their efficiency to chelate elemental iron of varying dynamic oxidation states to mitigate associated clinical conditions. Crit Rev Food Sci Nutr. 2022 Aug 9;1–27. 10.1080/10408398.2022.2106936. 35943179

[100] Zeng X, An H, Yu F, Wang K, Zheng L, Zhou W, et al. Benefits of iron chelators in the treatment of Parkinson’s disease. Neurochem Res. 2021 May;46(5):1239–51. 10.1007/s11064-021-03262-9. PMC8053182 33646533

[101] Jansová H, Šimůnek T. Cardioprotective potential of iron chelators and prochelators. Curr Med Chem. 2019;26(2):288–301. 10.2174/0929867324666170920155439. 28933303

[102] Kruszewski M. The role of labile iron pool in cardiovascular diseases. Acta Biochim Pol. 2004;51(2):471–80. 15218543

[103] Bi Y, Chen J, Hu F, Liu J, Li M, Zhao L. M2 macrophages as a potential target for antiatherosclerosis treatment. Neural Plast. 2019 Feb 21;2019:6724903. 10.1155/2019/6724903. PMC6409015 30923552

[104] Rudijanto A. The role of vascular smooth muscle cells on the pathogenesis of atherosclerosis. Acta Med Indones. 2007 Apr-Jun;39(2):86–93. 17933075

[105] Mikulska-Ruminska K, Anthonymuthu TS, Levkina A, Shrivastava IH, Kapralov AA, Bayır H, et al. NO˙ Represses the Oxygenation of Arachidonoyl PE by 15LOX/PEBP1: Mechanism and Role in Ferroptosis. Int J Mol Sci. 2021 May 17;22(10):5253. 10.3390/ijms22105253. PMC8156958 34067535

